# Construct Validity and Reliability of a Virtual Reality Cricket Batting Simulator

**DOI:** 10.1002/ejsc.70161

**Published:** 2026-03-19

**Authors:** Oliver R. Runswick, Mark Phillips, Jazmin Morrone, Jamie S. North, Luke Wilkins

**Affiliations:** ^1^ Department of Psychology, Institute of Psychiatry, Psychology & Neuroscience King's College London London UK; ^2^ Cover Driver Cricket Ltd Oxford UK; ^3^ School of Sport, Exercise and Applied Science St Mary's University London UK; ^4^ Department of Earth Science and Engineering Imperial College London London UK; ^5^ Sport, Performance, and Nutrition (SPAN) Research Group La Trobe University Melbourne Australia

**Keywords:** cricket, extended reality, motor skill, reliability, validity, virtual reality, VR, XR

## Abstract

Virtual reality technologies offer sports performance organisations new possibilities for measuring and enhancing performance. This is particularly the case in cricket where training is significantly constrained by weather and seasons, but game understanding is vital to high‐level performance. Before implementation, new simulations need to be tested for construct validity to ensure the virtual environment accurately represents the real‐world performance setting, and for reliability to ensure that improvements in performance can be accurately measured over time. Therefore, this study aimed to test the construct validity and reliability of a commercially available virtual reality cricket batting simulator. To test construct validity, we compared performance in the simulator across novice, club level, and elite performance pathway (EPP) cricket players. To test reliability novice and club participants completed the test four times across two separate days. Results supported construct validity through detecting differences in runs scored (*p <* 0.001) and wickets (*p =* 0.032) lost between the groups, however EPP and club players lost considerably more wickets than would be expected. Strong correlations and low bias between visits showed within‐ and between‐day agreement and reliability. Results support the suitability of this VR simulator in measuring runs scored in cricket batting but suggests players may take more risks and lose more wickets in a virtual setting.

## Highlights

- Evaluated a commercially available VR cricket batting simulator (Cover Drive Cricket) using a standardised five‐over (30‐ball) test with a real bat and Meta Quest 2 tracking, comparing 15 novice, 15 club, and 15 elite performance‐pathway (EPP) batters.
- Broad construct validity was supported: groups differed in runs scored (*p* < 0.001) and wickets lost (*p* = 0.032); EPP players scored more runs and lost fewer wickets than novices, with large (but non‐significant) effects between club and EPP.
- Test‐retest reliability was strongest for runs scored: performance was consistent within‐ and between‐day with minimal bias. Reliability for wickets lost was also acceptable, but less consistent across days.
- Notably, club and EPP players lost more wickets than expected; this may reflect tracking/physics limitations or altered decision‐making in VR (lower ‘cost of failure’), suggesting runs scored may be a more robust assessment outcome than dismissals.

## 1 Introduction

Virtual reality (VR) technology is becoming an increasingly popular tool for understanding and enhancing performance in sport (Richlan et al. 2023). While many definitions of VR exist, this paper will adopt the position that VR refers to ‘the simulation of an interactive three‐dimensional environment that users can be immersed into and that they can interact with’ (Sagnier et al. 2020, 993). This definition emphasises the fully immersive and interactive aspects of VR that distinguish it from adjacent technologies (such as mixed reality, augmented reality, and 360‐degree video) that exist within the XR spectrum (Le Noury et al. 2022; Runswick 2023) and which, importantly, are integral within the real‐world domain of sports.

VR enables practitioners to investigate the mechanisms underlying skilled performance by creating a representative environment that can be systematically manipulated and rigorously controlled (Wilkins 2024). VR can also be used to train athletic skills, particularly due to its ability to incorporate evidence‐based principles within the generated virtual environment (Gray 2019). In cricket, VR offers solutions to specific issues faced in the game. Training in match situations is logistically challenging and constrained by the weather but understanding game contexts are of vital importance to performance in the sport (Runswick et al. 2018, 2019), and specific populations often have reduced opportunities to be exposed to match play (Runswick et al. 2024).

Whether using VR to measure or enhance performance, for it to be effective, there needs to be strong transfer to the real world, which is dependent upon having acceptable levels of fidelity and construct validity (Wood et al. 2021). Fidelity refers to the degree of realism created by the virtual environment and includes physical, emotional, biomechanical, and psychological components, while construct validity refers to the degree to which task performance and behavior is accurately elicited by the virtual environment (Harris et al. 2021). Cricket‐specific VR simulators have been tested for psychological fidelity using questionnaire‐based measures and showed promising responses for highly skilled athletes (Runswick 2023). However, there is a lack of evidence to determine whether this translates to construct validity and the ability to measure or improve performance consistently.

Construct validity is commonly established via one of two methods. One approach involves having the same group of participants complete an equivalent task in both a VR environment and a real‐world environment, with construct validity supported if performance or behavior does not significantly differ (‘Within‐Subjects Approach’). The second approach compares the VR task performance of two groups with known differences in real‐world expertise, with construct validity inferred if the expected group differences are observed in the VR task (‘Between‐Subjects Approach’).

A recent study by Egiziano et al. (2025), adopted the within‐subjects approach to examine the construct validity of a custom‐built VR athletics relay simulator. Twelve French national‐level relay athletes completed eight trials of a relay running initiation task in both a VR and real‐world environment, with performance measured via three key event markers (wave initiation, gaze release, and foot lift). Strong support for the construct validity of the system was found, with the two conditions producing comparable key event sequencing as well as similar reductions in variability between trials. Taking the same approach to construct validity was the work by Pastel et al. (2025). Though their measures were not specific to a particular sport, they assessed motor skills integral to many; namely, reaction time, jump height, and agility. The performance of these skills in a VR environment and the real‐world by 32 participants found mixed support for the construct validity of the tests. Specifically, for reaction time and agility, performance significantly differed between the two conditions, though significant correlations were found, suggesting that the tests reflected relative validity but not absolute validity. The test of jump height showed a non‐significant difference and significant correlation between conditions, inferring good construct validity.

Adopting a within‐subjects approach to establishing construct validity in VR systems designed for team sports can be challenging due to the complexity of the key environmental features and greater number of interactive elements. The desire to include teammates and opponents, in particular, makes it difficult to design controlled and repeatable tasks in the real‐world. In such instances, a between‐subjects approach may be preferred. For instance, Wood et al. (2021), tested the ‘Rezzil’ VR platform for soccer with 17 elite, 17 elite‐youth, and 17 novice players. The elite players showed significantly greater performance than both the elite‐youth and novice players on three out of four tests, as well as on an overall diagnostic score designed to encapsulate additional aspects of performance during the tests. Interestingly, the elite‐youth only outperformed the novices on the diagnostic score, which the authors suggest may have been due to age‐related differences. Thusly, good, but not complete, support for the construct validity of the VR platform was found. Finally, Harris et al. (2021) utilised both approaches when testing the construct validity of a non‐commercial VR golf putting system. Here, 18 expert amateur golfers (mean handicap of 1.7) and 18 novice golfers (no handicap) were compared on a putting task with a 305 cm distance (Harris et al. 2021). The results provided support for the construct validity of the VR system as the expert group significantly outperformed the novice group by an average distance error of 30 cm in the VR condition, while there was a significant, positive correlation found between performance in the VR and real‐world conditions.

Alongside the establishment of validity and fidelity, new tools must also demonstrate acceptable levels of reliability, defined as ‘the ability of an instrument to consistently measure an attribute’ (DeVon et al. 2007, 160). This can be investigated through ‘test‐retest’, where the same group of participants complete a task at multiple time points. Reliability is inferred if performance remains sufficiently similar, assuming no influencing factors occur between tests. These can be conducted within the same day and between multiple days to ensure reliability in short and longer timescales (Vine et al. 2022; Runswick et al. 2021). Failure to establish reliability can lead to erroneous results and interpretation of findings, as the data obtained may be due to transient factors within the participant (e.g., fatigue or mood) or random error due to technological variations, rather than reflecting true performance (Atkinson and Nevill 1998). The previously mentioned work by Pastel et al. (2025) found large test‐retest correlations for their VR reaction time, jump height, and agility tests, indicating high reliability, while reliability has also been established in other sports‐related VR tools such as ones assessing concussions (Horan et al. 2020), reaction time (Polechoński and Langer 2022), and range of motion (Gumaa et al. 2021).

The present study had two aims: (1) examine the construct validity of a VR cricket batting simulator by comparing batting performance (runs scored and wickets lost) between players from three levels of expertise (novice, club‐level, and elite performance pathway; EPP), and (2) examine the reliability of the same VR system by having players perform the same task multiple times within the same day and on two separate days. It is hypothesised that performance will significantly differ according to expertise (elite being the best, novice being the worst), providing support for the construct validity of the VR system. This will be displayed by better players scoring more runs and losing fewer wickets. In addition, novice and club‐level participants will complete the batting test on four occasions: twice at visit one and twice at visit two, to provide data on within‐ and between‐day reliability. Such evidence is essential in assuring the efficacy of the tool before further use in both applied and academic settings.

## 2 Methods

### 2.1 Participants

A priori power analysis was conducted using G*Power 3.1 (Faul et al. 2007) to determine the required sample size for a one‐way ANOVA with three independent groups. This was based on the effect size for overall performance differences in VR between novice and academy footballers (*d* = 0.98) from Wood et al. (2021) as this best represented the existing evidence for our novice and EPP groups. *α* (alpha) was set at 0.05 and power (1 ‐ β) at 0.80. The total sample size required to detect a statistically significant difference amongst the three groups was calculated to be 44 participants. Forty‐five participants were recruited to form three evenly sized groups: 15 male club players (aged 27 ± 10 years) defined as individuals with experience as a batter, all‐rounder or wicket keeper playing at a competitive level (e.g., clubs or university team); 15 male elite performance pathway (EPP) players (aged 16 ± 1 year) from the development program of an English county who players as a batter, all‐rounder or wicket keeper; and 15 male novice participants (aged 32 ± 8 years) who were individuals that had not played beyond a recreational level (e.g., compulsory classes at school). All participants completed informed consent, including parents or guardians for players under 16 years old. Ethical approval was granted by the first author institutional ethics committee (LRS/DP‐20/21‐24513).

### 2.2 VR System Setup

The virtual reality system used was the Meta Quest 2 (Meta Platforms, USA), a standalone head‐mounted display with a single fast‐switch LCD and a horizontal field of view of ∼97°. The headset provides a display resolution of 1832 x 1920 pixels per eye and supports 90 and 120 Hz refresh‐rate modes. Processing is provided by a Qualcomm Snapdragon XR2 platform, and six‐degrees‐of‐freedom head and controller tracking is achieved via an inside‐out optical tracking system using four integrated headset cameras. Two standard Oculus Touch (third generation) handheld controllers are supplied with the device.

Cover Drive Cricket is a commercially available cricket batting simulation that allows users to control the types of bowlers they face, pitches used, and field settings to support targeted training. Participants hold a real cricket bat with a single Meta Quest 2 handheld controller attached rigidly to the shoulder of the bat, maintaining the six degrees of freedom tracking of the bat via the headset's tracking system. Independent bench testing of Meta Quest 2 controllers has reported translation standard deviations ranging from 1 to 14 mm across axes and rotational standard deviations ranging from 0.06° to 0.34°, indicating millimetre‐level positional precision in controlled setups (Pereira et al. 2023). The controller trigger is pressed when the player is ready to face a delivery, but no other buttons are required to play. The bat is used to strike the ball as in a real‐world game situation.

**FIGURE 1:**
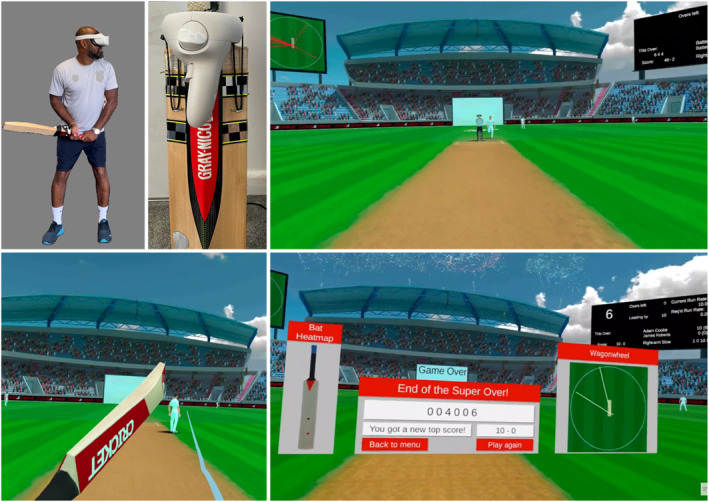
Images of the VR cricket simulation. Top left: set up of cricket bat and VR controller attached. Top right: virtual environment as seen by players before each delivery. Bottom left: screenshot immediately post bat‐ball contact; white trajectory line shows the path of the ball. Bottom right: feedback received by players at the end of each over.

Following each delivery, the simulation provides immediate auditory and visual feedback. When a bat‐ball contact was detected, a short audio clip approximating the sound of a wooden bat striking a hard ball was played through the headset speakers. The post‐contact trajectory of the virtual ball was determined by the game engine (Unity 2020.3, default physics engine), which updated the ball's position and velocity at 200 Hz (physics calculations in discrete timesteps of 0.005 s). Ball speed and direction after contact were calculated from the bat‐ball collision parameters and were dampened as a function of the contact point on the simulated bat (i.e., ‘middle’ vs. edges/shoulder) to represent more and less effective contacts. Depending on the quality and direction of the strike, and locations of fielders, players receive runs for each shot but are not required to run between the wickets themselves. For each shot, the game engine computes the earliest interception time for every virtual fielder based on their starting position, a fixed running speed, and the 3D position and velocity of the ball. The time between bat‐ball contact and the earliest interception (or, if no interception occurs, the time taken for the ball to reach the boundary) is then used to determine the number of runs awarded for that shot. As a result, shots that travel into gaps or over/between fielders create a longer time window before interception and yield more runs, whereas shots hit directly toward a nearby fielder result in fewer or no runs. Figure 1 shows images of the protocol setup and VR environment.

For this study, a five‐over simulation was created that used a ‘normal’ pitch type (i.e., average levels of bounce and deviation) and five bowler types (right‐arm slow, right‐arm medium‐fast, left‐arm slow, left‐arm medium‐fast, right‐arm leg spin). Fielders were placed in locations agreed upon with a panel of cricket coaches for each bowler type. The bowlers delivered a range of deliveries that were consistent in each test but delivered in a random order to avoid the task becoming predictable. This means all players faced the same 30 deliveries in every test. Participants were instructed to score as many runs as possible whilst losing as few wickets as possible in the five‐over test. The number of runs scored and the number of wickets lost were recorded for every test.

### 2.3 Procedure

Testing took place in a sports hall for EPP players and in a large lab space for the other groups, all of which allowed participants to move freely. After being instructed about the study and signing consent, participants put on a Meta Quest 2 headset and familiarised themselves with VR using the Meta First Steps app, which teaches participants how to operate in VR and use controllers. However, participants were deliberately not familiarised with the cricket simulation. This was to ensure participants did not become accustomed to the game, and the test captured how well the simulation represented the real sport rather than familiarisation with the VR game. Participants in the Novice and Club groups completed the two test visits separated by 48 h, with the interval identical for all participants. The EPP players completed the test once during a scheduled training session. The club and novice players completed the five‐over test four times; twice in visit one and twice in visit two.

### 2.4 Data Analysis

#### 2.4.1 Construct Validity

This analysis included only the first five‐over test to allow for the comparison between novice, club, and EPP group performance. One‐way ANOVA (three groups) was conducted for each of the two dependent variables (Runs, Wickets Lost). A Bonferroni adjustment was employed when multiple comparisons were being made to avoid Type I errors (McLaughlin and Sainani 2014). Violations of sphericity were corrected for by adjusting the degrees of freedom using the Greenhouse Geisser correction when epsilon was less than 0.75 and the Huynh‐Feldt correction when greater than 0.75 (Girden 1992). Cohen's *d* effect sizes were used for post hoc group comparisons, partial eta squared (*η*_*p*_^2^) for ANOVA analyses. The alpha level (*p*) for statistical significance was set at 0.05.

### 2.5 Reliability

The data from the Novice and Club groups across visit 1 test 1, visit 1 test 2, visit 2 test 1 and visit 2 test 2 were used to assess within‐day reliability. For between‐day reliability, scores from tests 1 and 2 within each visit were averaged to create a single visit 1 score and a single visit 2 score. Averaging multiple trials in this way provides a more stable estimate of typical performance and reduces the influence of random within‐session variability. Pearson's correlation and intra‐class correlation coefficients (ICC) were conducted. To determine agreement between sessions, Bland‐Altman analyses were conducted. Bland‐Altman (or difference plots) are a graphical method for comparing two different measurements and evaluating agreement through calculating and displaying both individual data points and a bias (average discrepancy between two trials or visits). Limits of agreement (LoA) display a range within which 95% of repeated measures would lie when compared to the first measurement. Effect sizes were calculated for all analyses; correlation coefficients (*r*) for Pearson's correlations and ICCs were accompanied by a 95% confidence interval. The alpha level (*p*) for statistical significance was set at 0.05.

## 3 Results

### 3.1 Construct Validity

#### 3.1.1 Runs

**FIGURE 2:**
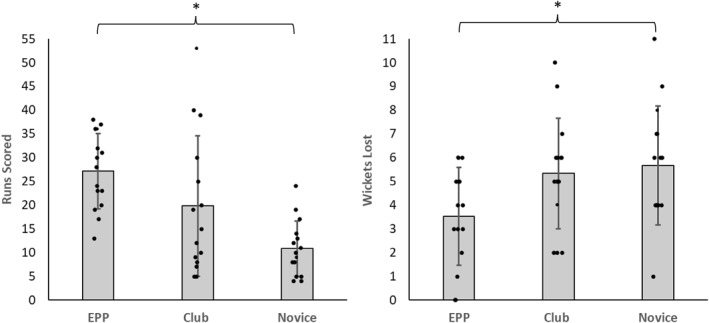
Scatter‐bar plots showing runs scored (left), wickets lost (right) in the first five‐over trial for novice, club, and elite performance pathway (EPP) players. Gray bars display means, error bars show standard deviation, dots show individual player data. *p < 0.05.

There was a significant main effect of Group on runs scored (*F* (2,42) = 9.485, *p* < 0.001, *η* ^*2*^ *p* = 0.311, Figure 2). Post hoc analysis showed that the EPP players (27.133 ± 7.945) scored significantly more runs than the Novice group (10.867 ± 5.829, *d* = 1.588, *p* < 0.001). The difference between Club (19.800 ± 14.756) and Novice was not significant but showed a large effect (*d* = 0.872, *p* = 0.065), as did the difference between Club and EPP (*d* = 0.716, *p* = 0.170).

#### 3.1.2 Wickets

There was a significant main effect of Group on wickets lost (*F* (2,42) = 3.731, *p* = 0.032, *η* ^*2*^ *p* = 0.151, Figure 2). Post hoc analysis showed that the EPP (3.533 ± 2.066) lost significantly fewer wickets than the Novice group (5.667 ± 2.498, *d* = 0.927, *p* = 0.045). The difference between Club (5.333 ± 2.320) and Novice was not significant (*d* = 0.145, *p* = 1.000), nor was the difference between Club and EPP, but there was a large effect (*d* = 0.782, *p* = 0.114).

### 3.2 Reliability and Agreement

#### 3.2.1 Runs Scored

**FIGURE 3:**
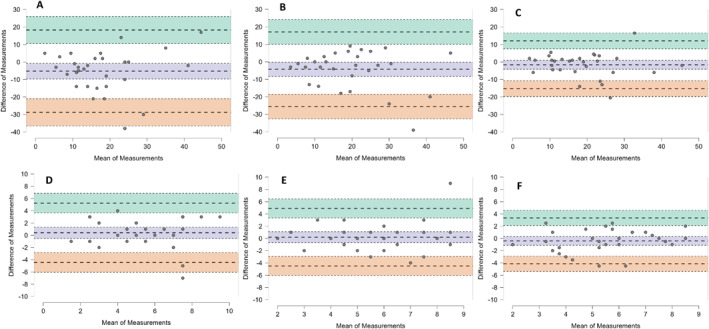
Bland‐Altman plots showing bias and limits of agreement (exact values are reported in text). The top row shows runs scored within‐day on (A) trial one and trial two (B) trial three and trial four and between‐day on (C) visit one and visit two. The bottom row shows wickets lost within‐day on (D) trial one and trial two (E) trial three and trial four and between‐day on (F) visit one and visit two.

The full descriptive data used for reliability and agreement analysis can be found in Table 1. Strong relationships and ICCs were found between tests one and two on the same day at visit one (*r* = 0.461, *p* = 0.005, ICC = 0.460, 95% CI = 0.126–0.701), between tests three and four on the same day at visit two (*r* = 0.582, *p* < 0.001, ICC = 0.573, 95% CI = 0.273–0.771) and between days on visits one and two (*r* = 0.767, *p* < 0.001, ICC = 0.765, 95% CI = 0.564–0.881). Figure 3 (top row) shows Bland‐Altman plots for test one and two on day one (Bias, 95% Limits of Agreement; −5.233, −9.714–−0.753), test three and four on day two (−4.233, −8.296–−0.170) and between visits one and two (−1.600, −4.198–0.988).

**TABLE 1:** Shows the mean (standard deviation) runs scored and wickets lost in each five over trial across the two visits for the club and novice players. The data from all 30 participants was used in reliability and agreement analysis.

	Visit 1 trial 1		Visit 1 trial 2		Visit 1 total		Visit 2 trial 1		Visit 2 trial 2		Visit 2 total	
	Runs	Wickets	Runs	Wickets	Runs	Wickets	Runs	Wickets	Runs	Wickets	Runs	Wickets
Club	19.80 (14.76)	5.33 (2.32)	26.60 (8.91)	4.93 (1.98)	23.20 (9.75)	5.13 (1.83)	21.93 (10.05)	5.53 (2.13)	24.93 (12.40)	6.20 (1.82)	23.43 (10.02)	5.87 (1.78)
Novice	10.87 (5.83)	5.67 (2.50)	14.53 (10.04)	5.27 (3.06)	12.70 (6.91)	5.47 (2.44)	12.93 (9.52)	6.07 (2.40)	18.40 (12.74)	5.00 (2.00)	15.67 (9.64)	5.53 (1.76)
All	15.33 (11.92)	5.50 (2.37)	20.57 (11.17)	5.10 (2.54)	17.95 (9.87)	5.30 (2.12)	17.43 (10.65)	5.80 (2.25)	21.67 (12.79)	5.60 (1.98)	19.55 (10.44)	5.70 (1.74)

#### 3.2.2 Wickets Lost

Medium‐to‐strong relationships and ICCs were found between tests one and two on the same day at visit one (*r* = 0.495, *p* = 0.003, ICC = 0.497, 95% CI = 0.172–0.724), between tests three and four on the same day at visit two (*r* = 0.361, *p* = 0.025, ICC = 0.370, 95% CI = 0.017–0.641) and between days on visits one and two (*r* = 0.525, *p* = 0.001, ICC = 0.518, 95% CI = 0.199–0.737). Figure 3 (bottom row) shows Bland‐Altman plots for test one and two on day one (Bias, 95% Limits of Agreement; 0.400, −0.523–1.323), test three and four on day two (0.200, −0.696–1.096) and between visits one and two (−0.400, −1.113–0.313).

## 4 Discussion

The present study aimed to examine the construct validity and reliability of a VR cricket batting simulator for measuring batting performance, with the goal of establishing the efficacy of the tool before its further use in both applied and academic settings. There was a significant main effect of group on batting performance, with higher skilled‐players (EPP) outperforming novice players. However, differences between the EPP and the club and novice groups did not reach statistical significance in post hoc tests but did show large effect sizes. This pattern indicates that the simulator can discriminate between broad skill levels and provides preliminary evidence of construct validity. However, the number of wickets lost in the test was higher than expected in the EPP and club groups. The reliability results suggested strong correlation and low levels of bias both within‐ and between‐days, suggesting that set tests in a VR simulation can offer a reliable measure of batting performance.

The ability to differentiate between skill level groups based on runs scored supports previous research from other sports that have shown construct validity for virtual simulations in head‐mounted displays (Egiziano et al. 2025; Harris et al. 2021; Wood et al. 2021). It also aligns with theoretical work, such as the concept of Representative Experimental Design. The nature of virtual *reality* is such that it should accurately represent the key features of an environment necessary for sports performance. Representative Experimental Design posits that the greater the correspondence between the real‐world environment and the experimental design (or, in this context, the virtual environment), the more accurate the results obtained will be in reflecting real behavior (Araújo et al. 2007). Moreover, Representative Experimental Design underpins the assumptions of Hadlow et al. ’s (2018) Modified Perceptual Training Framework, which predicts VR to be one of the most effective technologies currently available in sports training precisely because of its high degree of stimulus and response correspondence. In the present study, runs scored differentiated novice players from EPP players, though differences between the EPP and club were not significant. These findings offer preliminary support for these theoretical frameworks and suggests that the VR cricket batting simulation has potential utility as a tool for assessing batting performance at a broad skill level, although further research is needed before its validity can be firmly established.

A finding that warrants further investigation is the number of wickets lost. No significant differences were found between the EPP and club players, with both losing more wickets than would be expected. It is possible that this is due to software or hardware limitations such as tracking inaccuracies between the physical bat and virtual ball. Any systematic discrepancy between the real bat movement and the simulated bat‐ball interaction would have important implications for construct validity and for the biomechanical and psychological fidelity of the environment. More broadly, our construct validity assessment evaluates the VR system as an integrated whole, such that any small, residual tracking inaccuracies are already embedded in the observed performance differences. However, larger or systematic tracking errors could still distort performance outcomes in ways that undermine fidelity and validity. Dedicated calibration and validation work is therefore needed to quantify tracking accuracy and its influence on key performance outcomes in this and similar VR systems.

Alternatively, it may be that players bat more aggressively in the virtual setting than they would in the real world and are therefore losing their wicket more often. Whilst players were instructed to score as many runs as possible whilst losing as few wickets as possible (the premise of batting in cricket), the absence of meaningful consequences for failure may have influenced their approach. Both performance outcomes (runs scored and wickets lost) depend on perception‐action coupling, anticipatory skill, and decision‐making. A wicket in cricket is typically the result of a failed shot, just as scoring runs reflects successful shot execution. However, these outcomes may differ in their relative sensitivity to different aspects of fidelity. Runs scored represent the accumulation of successful bat‐ball contacts across many deliveries and are likely constrained primarily by the quality of perceptual information and the ability to coordinate the bat with the ball (i.e., psychological and biomechanical fidelity; Harris et al. 2020). By contrast, wickets lost are discrete failures that, in real match play, carry substantial emotional and competitive cost. This ‘cost of failure’ is closely related to affective fidelity—the extent to which a simulation elicits emotional and motivational states comparable to those in the real task (Harris et al. 2020). If dismissal in VR does not feel as consequential as dismissal in a real match, players may adopt more risk‐seeking performance strategies. This could artificially inflate wicket losses relative to real‐world performance without necessarily indicating poorer perceptual‐motor fidelity. We therefore tentatively suggest that runs scored may be more strongly influenced by psychological and biomechanical fidelity, whereas wickets lost may be particularly sensitive to affective fidelity, although direct measurement of affective responses and risk‐taking in VR versus real cricket is needed to test this hypothesis. Moreover, it is unknown whether adopting such risk‐taking strategies in VR negatively impacts skill acquisition (potentially due to practise becoming less representative of competition) or positively impacts skill acquisition by encouraging greater exploration of action affordances (Gray 2025).

As well as establishing some level of construct validity for the use of VR in cricket, we also assessed within‐ and between‐day reliability of scores. This is a vital piece of information if VR platforms are to be used for applications such as talent identification and performance testing. However, previous work on applications of VR in sport have lacked attempts to test this. Here we showed that a simple five‐over (30 delivery) test was able to produce low bias for runs scored between tests on the same day, and when combined over multiple visits offered a reliable measure of batting performance. The opportunity to conduct controlled testing—where all players face identical opponents and scenarios—in order to support selection decisions or accurately track skill development has been reported as one of the most important factors for implementing VR by applied practitioners (Dowsett et al. 2023). This could be particularly useful for cricket batting, given the substantial impact that variations in bowlers and pitch conditions can have on batting performance, as well as the logistical challenges introduced by inclement weather. Furthermore, VR is considered uniquely suited to the measuring of perceptual‐cognitive skills (Kittel et al. 2024) such as anticipation and decision‐making, which are crucial in cricket (Runswick et al. 2018; Marshall et al. 2024).

While the establishment of construct validity of this type is important in providing confidence for the use of VR in applied settings, future work must investigate the *extent* to which VR platforms can distinguish between expertise levels. Demonstrating that elite cricket batters outperform novices can be considered as *broad* construct validity, given the large real‐world differences in skill level (Wilkins and Middleton 2025). However, for VR to have potential use as a talent identification tool, more fine‐grained construct validity is needed. For such cases, VR platforms should be able to, at the very least, discern between elite and sub‐elite athletes—an ability that the present study may have achieved for runs scored were a larger sample recruited, but was far from attaining for wickets lost. Future studies may also wish to systematically examine features of VR platforms that contribute to increased or decreased construct validity. As mentioned previously, attempting to isolate and influence the physical, psychological, affective, and/or biomechanical fidelity of a platform would be of interest both theoretically, and to VR developers and practitioners wishing to optimise their virtual environments. New methods such as the development of wireless dry‐electrode EEG for use during a dynamic psychomotor VR task could support the development of nuanced validity investigation in sport‐related contexts (Morrone et al. 2025).

The findings presented in this study should be considered in light of its limitations. A primary issue in this work was that the highest‐level group of performers (EPP) were significantly younger than the club and novice groups. This issue may limit the expected performance differences, where some of these high performing young players may go on to play the same level of cricket as some of the club group and is a problem that has been evident in other VR validity work (Wood et al. 2021). This age difference could also offer some explanation for the high number of wickets lost. Adolescents in general are prone to higher levels of risk taking (Willoughby et al. 2021) and whilst risk taking in VR certainly warrant further investigation, this could offer some explanation. Whilst the EPP players offered access to a selective high‐performance population, they could not take part over multiple days, meaning reliability data only focuses on club players and novices. Whilst it could be important to investigate reliability in higher level players, performance is likely to be more consistent in higher level players.

## 5 Conclusion

The present study compared the performance of novice, club‐level, and EPP cricket players on a VR batting task, and assessed the reliability of that performance over multiple test sessions. The EPP group outperformed novices in both runs scored and wickets lost, establishing a degree of construct validity. Reliability and agreement results suggest that the VR test was able to reproduce performance consistently both within‐ and between‐days. The work conducted here offers a first step in understanding the use of VR in cricket batting, but, whilst a well‐established approach, comparing skilled to novice performers only offers a crude understanding of construct validity. Further work is required to understand the ability of VR simulations to differentiate players in a more fine‐grained fashion for applications such as talent testing where there is a need to measure and establish differences in performance within a more homogenous cohort of players.

## Author Contributions

**Oliver R. Runswick:** conceptualization, methodology, formal analysis, resources, writing – original draft, project administration, funding acquisition. **Mark Phillips:** investigation, data curation. **Jazmin Morrone:** investigation, data curation, writing – review and editing, project administration. **Jamie S. North:** investigation, data curation, writing – review and editing, project administration. **Luke Wilkins:** conceptualization, writing – original draft.

## Funding

The authors have nothing to report.

## Conflicts of Interest

Mr. Phillips is Director and Dr Runswick owns shares in Cover Drive Cricket Ltd., who are a virtual reality cricket company, and the app used in this paper.

## Data Availability

The data that support the findings of this study are openly available in OSFHome at https://osf.io/ya2pu/?view_only=8091af3ef11041be81673ba6950c8c23.
